# What Opinions Do Tumor Reconstructive Surgeons Have about Sports Activity after Megaprosthetic Replacement in Hip and Knee? Results of the MoReSports Expert Opinion Online Survey

**DOI:** 10.3390/jcm9113638

**Published:** 2020-11-12

**Authors:** Gerhard M. Hobusch, Florian Keusch, Hiroyuki Tsuchiya, Michael Joyce, Reinhard Windhager

**Affiliations:** 1 Department of Orthopaedic and Trauma Surgery, Medical University of Vienna, 1090 Vienna, Austria; reinhard.windhager@akhwien.at; 2 Department of Sociology, University of Mannheim, 68131 Mannheim, Germany; fkeusch01@gmail.com; 3 Department of Orthopaedic Surgery, Kanazawa University, Kanazawa 920-1192, Ishikawa, Japan; tsuchi@med.kanazawa-u.ac.jp; 4 Department of Orthopaedic Surgery, Cleveland Clinic, Cleveland, OH 44106, USA; joycemich@aol.com

**Keywords:** megaprostheses, recommendations, quality of life (QOL), tumor resection, revision surgery, rehabilitation

## Abstract

Sports activity has many benefits in cancer survivors. A key one is having sport activity contribute to the well-being of the individual. However, there are no guidelines about the intensity and kind of postoperative mobility workouts after hip or knee megaprosthetic treatment. Opinion research about sports after modular bone and joint replacement may provide an understanding of surgeons’ attitudes on sports activity after megaprostheses of the hip and knee joint. A web survey with members of three international professional organizations of orthopedic tumor reconstructive surgeons was conducted between September 2016 and January 2018. Members were invited via personalized emails by the European Musculoskeletal Oncology Society (EMSOS), the International Society of Limb Salvage (ISOLS), and the Musculoskeletal Tumor Society (MSTS). The questionnaire included 26 questions. A total of 149 surgeons started the survey, and 76 finished the entire survey (American Association for Public Opinion Research (AAPOR) second response rate (RR2) EMSOS: 12.3%; ISOLS: 21.9%; MSTS: n/a). More than half of the respondents encourage sarcoma survivors after megaprosthetic treatment to reach an activity level that would allow them to regularly participate in active sporting events of University of California, Los Angeles (UCLA) activity level 7 and higher. Orthopedic tumor reconstructive surgeons do fear a number of complications (periprosthetic fracture, allograft failure/fracture, loosening, prosthetic or bearing failure, and early polyethylene wear) due to sports activity after modular bone–joint replacement, but they actually witness fewer complications than they conceptually anticipated. According to the surgeons’ opinions, between four to seven types of sports after surgery could reasonably be recommended depending on the type of hip or knee procedures. This survey provides insights into opinions on what could be recommended, what could be allowed if surgeons and their patients agree on the potential negative outcome, and which sports should definitely not be allowed after hip and knee megaprostheses.

## 1. Introduction

Most public authorities perceive sports as an important societal benefit and include sports participation in their policies [1,2,3]. Sports is an important leisure activity with many benefits [4]. According to European community (EU) statistics, 61% of young people regularly participate in sports, and average sports activity is increasing in the general public [5,6]. However, some people are denied active participation in sports for various orthopedic reasons. This applies in particular to patients after orthopedic reconstructive surgery, including megaprosthetic replacement, partly because they are not able to resume or attain their preoperative sports activity levels after accidental or oncologic life events, partly because their physicians do not dare to endanger their orthopedic reconstructions. In this situation, people with physical impairment are often adapting to their loss of function; however, patients who were active in sports before an impairing life event and bound to lower activity level thereafter usually mourn this loss deeply [7].

To avoid patients’ loss of physical sport activity after knee and hip replacement, expert opinions about sports have been defined by knee and hip societies in order to make some sports possible after surgical treatment without harm [8]. Currently, there is scarcely information available about sports activity for the time after megaprosthetic replacement. Several recent case series including patients after bone and joint mega-replacement elucidated the immanent importance of sporting activities for these patients. However, years-long rehabilitation was necessary after an operation of this magnitude to finally reach moderate-to-high levels of sporting activity after replacement in the proximal and distal femur and in the proximal tibia [9,10,11].

There is not a uniform recommendation on whether sporting activity could be allowed after megaprosthetic replacement: while some tumor and adult reconstructive surgeons restrict routine total joint patients from even moderate athletic endeavors, others set the patient-expectations-in-achievement bar quite high [12]. A survey among orthopedic tumor surgeons, who regularly do lower-extremity bone tumor and megaprosthetic replacement in active younger patients, may help to define limits within sports that may be possible for patients after these kinds of treatment. Amongst other issues within this survey, the following questions needed to be raised and answered: What activity level do surgeons encourage sarcoma survivors to eventually reach after megaprosthetic bone–joint replacement? Which complications and failures due to sports activity do surgeons fear and actually witness after modular bone–joint replacement? What sports do surgeons recommend, allow, and not allow to sarcoma survivors after modular hip or knee lower-limb reconstructions?

## 2. Methods

To answer our research questions, we conducted a web survey with members of three international professional organizations of tumor reconstructive surgeons: the European Musculoskeletal Oncology Society (EMSOS), the International Society of Limb Salvage (ISOLS) and the Musculoskeletal Tumor Society (MSTS) between September 2016 and January 2018. The questionnaire comprised 26 questions and was programmed in Qualtrics Research Core. The target population were the members of the three organizations, and we used the membership lists of these organizations as the sampling frame for our study. At the time of first contact, the membership lists of the three organizations included 155 (EMSOS), 310 (ISOLS, excluding all EMSOS members), and 335 (MSTS) members.

Personalized e-mail invitations were sent to EMSOS members on 14 September 2016, with up to four follow-up reminders to nonrespondents on 21 September, 20 October, and 20 December 2016, as well as on 26 February 2017 (field period: 14 September 2016–6 April 2017). Personalized e-mail invitations were sent to ISOLS members on 4 May 2017, with up to three e-mail reminders for nonrespondents on 23 May, 4 July, and 18 August 2017 (field period: 4 May–30 August 2017). For MSTS members, we did not have access to individual e-mail addresses; thus, invitations were sent as part of the regular MSTS newsletter on 13 December 2017 with 2 reminders (field period: 13 December 2017–17 January 2018). There can be overlap since many members of MSTS and EMSOS are also members of ISOLS, and only a single response was recorded for an individual surgeon.

A total of 149 surgeons started the survey, and 76 completed the entire questionnaire (I = Interviews) with an additional 35 surgeons breaking-off after the demographic information (P = Partial Interviews) about half-way through the questionnaire. All analyses use *n* = 111 (I + P). Table 1 summarizes the participation behavior for members of the three organizations. The response rate as calculated based on the Standard Definitions by the American Association for Public Opinion Research [13] was 12.3% (AAPOR second response rate (RR2)) for EMSOS members and 21.9% for ISOLS members. We do not know how many MSTS members received the newsletter with the invitation to our survey; thus, we cannot calculate a response rate for this group. Since surgeons could be members in multiple organizations, our response rates are conservative estimates of the true response rate. The sociodemographic as well as regional characteristics of the respondents are presented in Table 2.

First, respondents were asked to indicate their agreement with two items about the influence of sporting activity on sarcoma survivors’ well-being on a five-point rating scale (strongly disagree–strongly agree) (see Section 3.1).

Second, they were asked about the encouraged activity level after surgery measured by using the 10-point University of California Los Angeles (UCLA) activity scale [14] (see Section 3.2).

**Figure 1:**
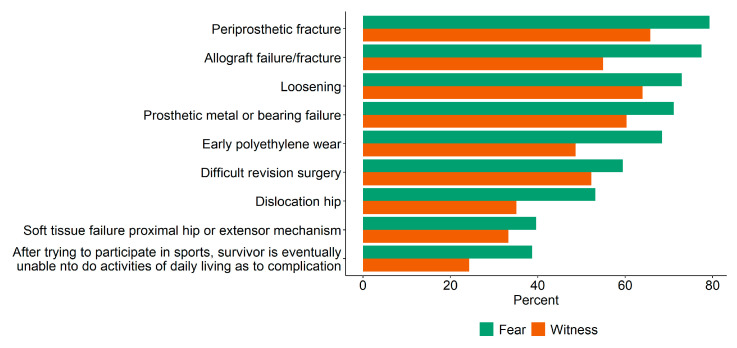
Percent of respondents who feared and witnessed post-surgery complications due to sports activity.

Third, respondents were asked which of nine sports-activity-associated complications and failures they fear after modular bone–joint replacement in sarcoma survivors and which of these they had actually witnessed (see Figure 1. The “feared” to the “witnessed” complications were compared conducting McNemar tests and calculating odds ratios (see Section 3.3).

Fourth, respondents were asked whether they would be more likely to allow a sarcoma survivor to participate in a sport after megaprosthetic replacement surgery if the survivor had prior experience in the particular sport. We then provided respondents a list of 40 different sports and asked the respondents which of the activities they would recommend, which of them they would allow if a survivor asked, and which of them they would not allow sarcoma survivors to do after megaprosthetic replacement surgery. These questions were asked for up to five different modular hip or knee replacement limb salvage procedures based on the surgeons’ experience with the procedure (see Section 3.4).

Finally, the recommendations for sports were compared on whether they differ by experience practicing tumor-orthopedic surgery (15 years or less vs. more than 15 years) using a Mann–Whitney U tests and by region (Europe vs. Americas vs. Asian Pacific) using a Kruskal–Wallis test for the comparisons of median number of sports recommended, allowed, and not allowed as well as chi square tests for the proportion of recommendations for individual sports (see Section 3.5).

All analyses were conducted in R version 3.6.1 [15], Dunn–Šidák correction was used for multiple comparisons. The sequence of the 40 sports corresponds to the average naming by participants according to recommended, recommended if asked, and not allowed (see Table 3).

## 3. Results

### 3.1. Attitudes toward Sporting Activity after Modular Bone–Joint Replacement

Almost half of the respondents (47%; 95% confidence interval (CI): 37%–57%) strongly agreed and 38% (95% CI: 29%–48%) somewhat agreed with the statement that sport participation is an important contributor to sarcoma survivors’ feelings of well-being after modular bone–joint replacement. Around 9% (95% CI: 5%–16%) neither agreed nor disagreed with the statement, and only 6% (95% CI: 3%–13%) somewhat disagreed or strongly disagreed. Similarly, 44% (95% CI: 35%–54%) of respondents strongly agreed and 44% (95% CI: 35%–54%) somewhat agreed with the statement that sport participation has a positive effect on survivors of bone sarcoma after modular bone–joint replacement. Less than 5% (95% CI: 2%–11%) neither agreed nor disagreed, and around 7% (95% CI: 3%–14%) somewhat or strongly disagreed with the statement.

### 3.2. Activity Levels Encouraged to Reach after Modular Bone–Joint Replacement

**Figure 2:**
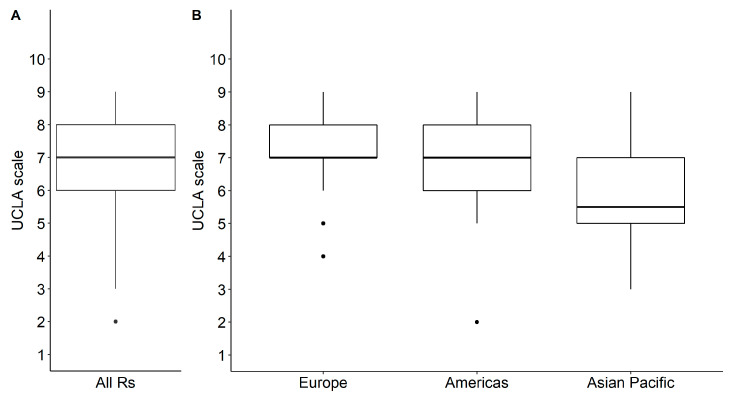
Box plot of responses to University of California, Los Angeles (UCLA) activity scale: (**A**) all respondents; (**B**) by region. UCLA Score: 1: Wholly inactive, dependent on others, and cannot leave residence; 2: Mostly inactive or restricted to minimum activities of daily living; 3: Sometimes participates in mild activities, such as walking, limited housework, and limited shopping; 4: Regularly participates in mild activities; 5: Sometimes participates in moderate activities such as swimming or could do unlimited housework or shopping; 6: Regularly participates in moderate activities; 7: Regularly participates in active events such as bicycling; 8: Regularly participates in active events, such as golf or bowling; 9: Sometimes participates in impact sports such as jogging, tennis, skiing, acrobatics, ballet, heavy labor, or backpacking; 10: Regularly participates in impact sports.

More than half of the respondents encourage sarcoma survivors to reach an activity level that would allow them to regularly participate in active sporting events after modular bone–joint replacement (UCLA activity scale median: 7; range 2–9) (see Figure 2).

### 3.3. Feared and Witnessed Post-Surgery Complications Due to Sports Activity

Respondents do fear a number of complications due to sports activity after modular bone–joint replacement, but they actually witness fewer complications (Figure 2). A majority of respondents feared periprosthetic fracture (79%; 95% CI: 70%–86%), allograft failure/fracture (77%; 95% CI: 68%–85%), loosening (73%; 95% CI: 64%–81%), prosthetic or bearing failure (71%; 95% CI: 62%–79%), and early polyethylene wear (69%; 95% CI: 59%–77%) as post-surgery complications from sports activity. The most frequently reported sport-activity-associated complications after surgery were periprosthetic fracture (66%; 95% CI: 56%–74%), loosening (64%; 95% CI: 54%–73%), and prosthetic or bearing failure (60%; 95% CI: 51%–69%). There was a discrepancy between what respondents reported to fear and what they actually witnessed for the four types of complications. Respondents were less likely to witness than to fear early polyethylene wear (odds ratio (OR) = 0.15; 95% CI: 0.03–0.37; *p* < 0.001), periprosthetic fracture (OR = 0.25; 95% CI: 0.05–0.60; *p* = 0.04), dislocation of the hip (OR = 0.15; 95% CI: 0.03–0.39; *p* = 0.02), and allograft failure/fracture (OR = 0.19; 95% CI: 0.06–0.41; *p* < 0.001).

### 3.4. Recommended, Allowed, and Not Allowed Sports after Modular Bone–Joint Replacements

**Figure 3:**
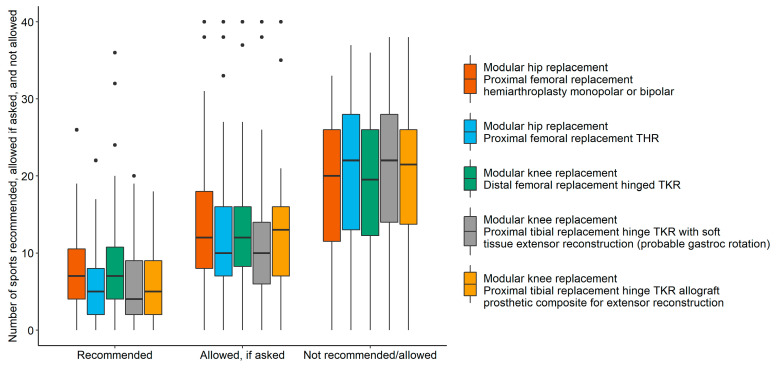
Number of recommended, allowed, and not allowed sports after surgery by procedure.

Sixty-four percent (95% CI: 51%–74%) of respondents reported being more likely to allow a patient to participate in a sport postoperatively if the patient had prior experience with this sport. Thirty-six percent (95% CI: 26%–49%) of respondents stated that it made no difference in recommendation when prior experience was evident. After proximal femoral replacement hemiarthroplasty, monopolar or bipolar, a median of seven (95% CI: 5–8) different sports were recommended by respondents; 12 (95% CI: 10–15) were allowed, if asked by the patient; and 20 (95% CI: 17–23) were not allowed (Figure 3). After proximal femoral replacement total hip replacement (THR), a median of five (95% CI: 3–7) sports were recommended; 10 (95% CI: 7–14) were allowed, if asked; and 22 (95% CI: 19–26) were not allowed. After distal femoral replacement hinged total knee replacement (TKR), a median of seven (95% CI: 5–8) sports were recommended; 12 (95% CI: 10–14) were allowed, if asked; and 19.5 (95% CI: 16–22) were not allowed, if asked. After proximal tibial replacement hinge TKR with soft tissue extensor reconstruction probable gastrocnemius rotation, a median of four (95% CI: 3–7) sports were recommended; 10 (95% CI: 9–13) were allowed, if asked; and 22 (95% CI: 19–26) were not allowed. After proximal tibial replacement hinge TKR allograft prosthetic composite for extensor reconstruction, a median of five (95% CI: 3–7) sports were recommended; 13 (95% CI: 10–15) were allowed, if asked; and 21.5 (95% CI: 16–26) were not allowed. Walking, swimming, and stationary bicycling were the most often recommended sporting activities after surgery (Table 3).

### 3.5. Differences in Recommendations by Regions and Experience

Recommendations about what activity level was encouraged and what sports were recommended, allowed, and not allowed differed by region but not by the years of experience practicing tumor-orthopedic surgery. Respondents from Europe (median = 7; range: 4–9; *p* = 0.003) and the Americas (median = 7; range: 6–9; *p* = 0.001) encouraged sarcoma survivors to reach a higher level of activity than did respondents from Asian Pacific region (median = 5.5; range: 5–9; Figure 2B). Only 29% (95% CI: 11%–56%) of respondents from Asian Pacific countries recommended golf after proximal femoral replacement hemiarthroplasty, monopolar or bipolar, compared to 71% (95% CI: 55%–84%; *p* = 0.008) of respondents from the Americas, and only 24% (95% CI: 8%–50%) of Asian Pacific respondents allowed hunting, if asked by the sarcoma survivor compared to 71% (95% CI: 55%–84%; *p* = 0.022) from the Americas. Respondents from Asian Pacific countries more often did not allow gymnastics (88%; 95% CI: 60%–98%) compared to European respondents (29%; 95% CI: 12%–52%; *p* = 0.034) after proximal femoral replacement THR. While only 43% (95% CI: 19%–70%; *p* = 0.032) of respondents from Asian Pacific countries recommended stationary bicycling, 89% (95% CI: 70%–97%) of respondents from Europe and 88% (95% CI: 72%–95%) of respondents from the Americas did so after distal femoral replacement hinged TKR. For the same procedure, fewer respondents from Asian Pacific countries (7%; 95% CI: 1%–34%) compared to European respondents (59%; 95% CI: 39%–77%; *p* = 0.047) recommended road bicycling. Finally, fewer European respondents (28%; 95% CI: 13%–50%) than respondents from the Americas (81%; 95% CI: 62%–92%) and Asian Pacific countries (92%; 95% CI: 60%–99%; *p* = 0.008) did not allow gymnastics after proximal tibial replacement hinge TKR with soft tissue extensor reconstruction with probable gastrocnemius rotation (Table 4).

## 4. Discussion

Megaprosthetic replacement is commonly performed in lower-limb reconstruction after tumor resections [16,17,18,19] as well as increasingly after trauma [20,21,22] or failed primary endoprosthetic replacement [23,24]. Despite their high rates of long-term sequelae including infection, mechanical and soft tissue failures, and loosening, patients benefit from surgery by being able to use their lower extremities in activities of daily living. However, once patients enter the rehabilitation phase after megaprosthetic replacement, mobility and function become vaguely described and patients as well as therapists may become confused as to possible mobility restrictions compared to practical endoprosthetic use. A recent systematic review about fitness, function, and exercise training responses after limb salvage with lower-limb megaprostheses concludes that exercise interventions are required. Interestingly, the authors describe the absence of outcome measure items assessing higher-level functioning [25]. Especially for young patients who are faced with a cancer diagnosis early in life, it is important to regain physical performance and mental and social well-being. Currently, sporting activities for handicapped people are facilitated both by creating opportunities such as organized disabled sporting events and by the provision of novel technical developments in exoprosthetics and bionics [26,27]. Of note, the importance of sports activity in oncologic disease has been widely reported with benefits in cancer cohorts [28].

This current survey was therefore assessed in orthopedic tumor societies expecting that surgeons are frequently confronted with requests for sporting activities by younger, active cancer survivors reconstructed with megaprostheses.

Oncologic as well as reconstructive surgeons seem divided over what degree (light activity to strenuous) of sporting activity should be allowed after megaprosthetic limb reconstruction. The most recommended sports were walking, swimming, and playing golf. Only 14% of the respondents allowed some impact-type activities, although nearly 2/3 of the participants were more likely to allow patients to participate in a sport postoperatively when it had been practiced before such as bicycling, ballroom dancing, and bowling, most respondents favored allowing the lower degree of a vigorous activity. Only 15% of the listed sports were recommended, but over 50% of the listed sports were not recommended at all. Most respondents may have considered proximal femoral replacement hemiarthroplasty, monopolar or bipolar, and distal femoral replacement the most stable reconstructions and, therefore, recommended a median of seven different sports. Five different sports were recommended by respondents after proximal femoral replacement THR and after proximal tibial replacement hinge TKR allograft prosthetic composite for extensor reconstruction. Only four different sports were recommended after proximal tibial replacement hinge TKR with soft tissue extensor reconstruction probable gastrocnemius rotation (Table 3). These recommendations may partly reflect the incidence of possible failures in these particular reconstructions (see later), but even more the fear for difficult re-reconstruction, worse functional outcome [29], and higher amputation rate especially after proximal tibia reconstruction [30]. In comparing proximal tibia reconstructions, there may be more stability and confidence in TKA allograft composite for extensor reconstruction than in TKA with soft tissue reconstruction in the respondents´ perception, which goes in line with data published by Müller et al. [31]. Hemiarthroplasty appears to have the lowest risk for instability in the proximal femur reconstruction than THR [32]. However, since recommendations on which sports can safely be done are not available and are often limited to the personal experience of the surgeon, even recommending some of these sporting activities (Table 2) may serve as a possible guideline in postoperative care of these patients.

Healy et al. reported similar sport restrictions in the first consensus expert opinion about sports activity after elective joint arthroplasty of the hip in 1999. In a second consensus paper 2005, several more sports were recommended that had previously been only just allowed [8,33]. The reason for the relaxations in recommendations after primary elective joint replacement might possibly be a change in attitudes of surgeons by observing more vigorous activities in their patients without witnessing any late complications. A more restrictive approach to recommending only some sports contradicts the recommended UCLA level of 7/10 when general activity was requested by the patient. Amstutz et al. defined a UCLA sports activity level of six after elective hip replacement as already high activity [14]. This discrepancy may elucidate once more the uncertainty of surgeons when recommending or allowing a particular sporting activity. There is the fear for a catastrophic event, which often prohibits allowing a more vigorous activity.

Megaprosthetic reconstruction bears the risk for failure in the long term. A comprehensive failure mode classification by the ISOLS serves as the mainstay in describing mechanical (aseptic loosening, soft tissue and structural component failure) but also non-mechanical failures. Authors of this multicenter study reported about 49% mechanical failures out of 534 failures in 2174 primary megaprosthetic replacements. A statistical dependence of anatomic region was found showing a total of 8% percent mechanical failures after proximal femur, 14% after distal femur, and 13% after proximal tibia megaprosthetic reconstruction [34]. According to Grimer et al., the vast majority of patients (82%) will need at least one revision surgery after 30 years following first-generation megaprosthesis [17]. In spite of modern megaprosthetic design changes, aseptic loosening and structural failure exist in 16%–19% of patients [35,36]. Furthermore, in oncologic cases, chemotherapy may affect osseointegration of megaprosthesis in the first 3 years, increasing the risk for aseptic loosening [37]. However, there is no clear evidence in the literature as to which activity levels lead to mechanical prosthetic failure or whether a higher activity level and exercise may control falls and even prevent from consequent prosthetic hardware failure [38]. Furthermore, there is hardly evidence that revision surgery worsens activities of daily living (ADLs) outcome in the long run. According to Biau et al. [18], body weight and activity levels were independent risk factors for early revision after megaprostheses around the knee but did not mention a specific activity loss after revision surgery. Hobusch et al. and Lang et al. reported possible events of loosening or structural failure in association with higher activity levels with proximal femur and around-the-knee megaprostheses. However, the patients’ subsequent activity level was not influenced by revision surgery [9,10,11]. Most surgeons in this current survey (71%–79%) seem more conservative because of fear of a second operation and patients never achieving the original first procedural function if too vigorous of sporting activity is undertaken and failure occurs. Furthermore, the high percentage of fear for sporting-activity-associated complications seem justified considering the high percentage of the reported witness of activity-associated failures. In fact, 60%–66% of the respondents witnessed periprosthetic fracture, loosening, or prosthetic or bearing failure in association with activity, and one quarter of the respondents even witnessed complication episodes in patients who were unable to resume activities of daily living (ADLs). Authors in musculoskeletal oncology literature usually report on Musculoskeletal Tumor Society Score (MSTS) or Toronto Extremity Salvage Score (TESS) outcome data and failure modes without reporting on both together [39]. There is a gap in knowledge about a relationship between sporting activities and failures as well as quality of life following megaprosthetic treatment.

The apparent surgeon expectations were more conservative than what was reported in published case-series unless patients requested to be allowed to do a more vigorous activity. The key point seems to be whether the surgeon is rigid in the initial recommendations or is flexible in allowing a more vigorous activity when the patient makes a request. The “fear” evidence as reported by the respondents suggests that it is appropriate not to encourage patients to overdo sporting activity beyond what is recommended in this current expert respondent review. However, once the patient asks for an increase in vigorous activity level and the surgeon is confronted in possibly modifying their recommendation, a shift of responsibility occurs by counseling the patient about the possible undesirable outcome. Surgeons are the ones who have to take care of the complications and hope to bring the patient back to their best possible ADLs. Interestingly, no respondent wanted to quantitate the recommended number of days of the week or length of the duration of activity in this survey.

**Figure 4:**
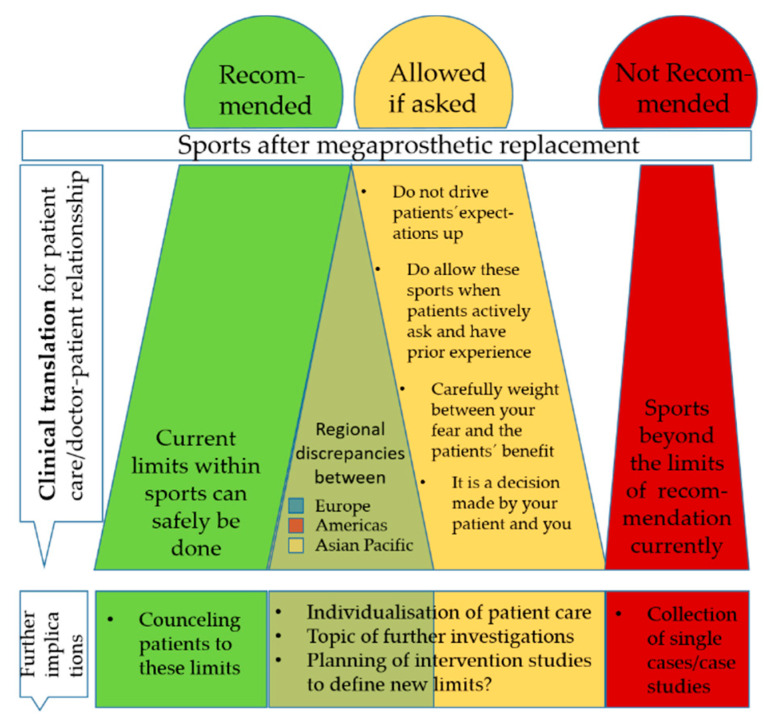
Graphical summary of sport recommendations after megaprosthetic replacement.

This survey ought to help define the conceptual limits between which sporting activities after megaprosthesis may be allowed. The types of sports, defined as recommended in this survey, may be safely done by the patients. Furthermore, the type of sports, defined as allowed if asked, ought to be subject of further investigations in this reconstruction field (see Figure 4).

According to the European Sports Charter, “Sports” not only means all forms of physical activity aimed at expressing or improving physical fitness and mental well-being but it is also means forming social relationships [40]. A cross-national study including 33 countries found that nearly 90% of individuals positively perceive sports as an arena for socialization [1]. This influence can also be turned in the other direction as different geographic regions form their specific sports attitudes. It therefore comes as no surprise that expert opinions concerning sports differ between these regions (see Table 4). Cross-continental differences in sporting habits may lead to different perceptions of certain sports and likelihood of allowing them or prohibiting the activity after lower-extremity limb salvage reconstruction. Surgeons may not be concerned about the reconstructed patient playing the most popular sports in their respective country when the surgeon is familiar with the activity. Examples would be playing golf in the United States or riding a road bike in Europe. The level of sporting activity should be balanced as to risks of complications from the level of activity and cultural norms. In some Asian Pacific regions, even regular joint replacement patients are not allowed for higher activity because of a dislike for revision prosthetic replacement surgery or for caution. The international differences in sports activity levels ought to be discussed in international panels to possibly create new approaches and equalities in international future sport recommendations.

Since this current online survey was done in musculoskeletal oncologic societies, issues of quality of life (QOL) were an initial primary focus. This current survey shows a broad agreement of 85% of orthopedic tumor surgeons on sporting activities as a contributor to well-being and even 88% who feel that there are positive effects of sport activity in survivors after bone and joint modular prosthetic replacement. These opinions reflect already existing literature about quality of life (QOL), mobility, and limitations in survivors of bone sarcoma and the obvious need to promote physical activity after bone tumor treatment [41]. Ness et al. reported a relative risk of 2.6–3.2 to develop performance limitations in bone sarcoma patients as well as a relative risk of 5.5–7.0 to develop restricted routine activities [42]. In line with these results, several authors reported the lowest physical functioning item of the Short Form 36 Health Survey Questionaire (SF-36) scores in survivors of bone sarcoma when compared with that in all cancer populations [43]. Furthermore, current preliminary data suggest an underlying association between physical activity and mortality for survivors of different cancers [44].

There are several limitations in this survey. First, the number of participating surgeons is low when measured in terms of orthopedic oncologic practice. The number of surgeons involved based on mean age and mean surgical experience seemed to be a representative group. This survey of surgeons assesses the individual biases of surgeons based on their anecdotal experience, as opposed to being evidence based. This survey does address whether a surgeon has “ever witnessed” the reported complication, which does not address the rate of the complication in the overall population or reconstruction-specific populations. Furthermore, in addition to intercontinental differences of recommendations, there might also be differences between European countries, which because of the low number from single countries are not significant according to this survey.

## 5. Conclusions

**Figure 5:**
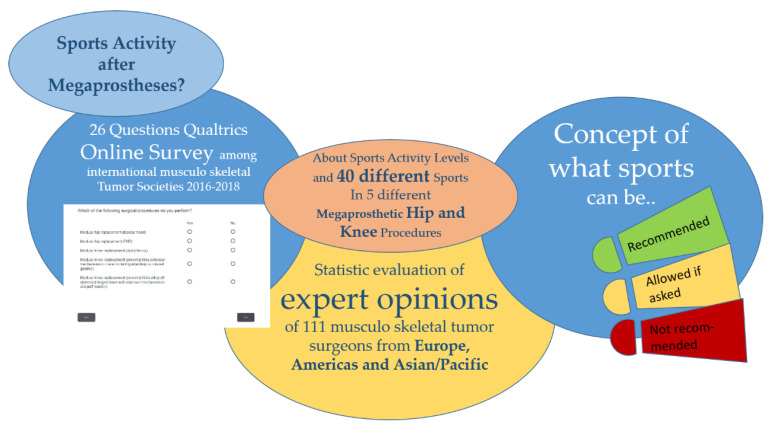
Graphical abstract of the Mo(dular) Re(placement) Sports Survey.

This survey provides insights into the current practice of surgeons counseling their patients toward sporting activities. Based on this knowledge, intervention studies may be planned to further translate current practice into recommendations (see Figure 5).

This survey ought to define the limits between which sporting activities after megaprosthesis may be seen to be safe. The types of sports, defined as recommended in this survey, may be safely done by the patients. There is consensus about the benefits of some light-impact sporting activity after lower-extremity limb salvage reconstructions.

Furthermore, the type of sports, defined as allowed if asked, ought to be the subject of further investigations in this important field. The fear of disaster and need to do another surgery is a realistic concern, and surgeons really have to ask the question as to why they compromise when patients request to do more vigorous sporting activity. It is important that the expectations of patients should not be driven upward to a higher sports activity level to their detriment, e.g., needing a second operation. However, once patients ask for certain activities in the gray area between recommended and not allowed, surgeons can only hope patients accept their “wishes within boundaries.” Individualization of a patient management may also need to include the patients’ personal wishes.

As such, this currently presented survey may represent an understanding of what other surgeons allow for postoperative sports activity after modular megaprostheses implantation. The survey provides some concept of what can be recommended, what can be allowed if surgeons and their patients agree on the potential negative outcome, or which sports should definitely not be allowed. Objective data about the relation of activity levels and prosthetic failure are needed.

## Figures and Tables

**Table 1:** Participation behavior and American Association for Public Opinion Research (AAPOR) response rates.

Disposition Codes	EMSOS	ISOLS	MSTS
Invitations sent	155	310	n/a
Survey started (I + P + R)	37	82	30
Completed entire survey (I)	19	46	11
Broke off after demographic Qs (P)	0	22	13
Broke off before demographic Qs (R)	18	14	6
Never reacted (U)	117	227	n/a
Bounced email	1	1	n/a
AAPOR RR1 (I/(I + P + R + U))	12.3%	14.8%	n/a
AAPOR RR2 ((I + P)/(I + P + R + U))	12.3%	21.9%	n/a

EMSOS = European Musculoskeletal Oncology Society; ISOLS = International Society of Limb Salvage; MSTS = Musculoskeletal Tumor Society; RR1 = first response rate; RR2 = second response rate.

**Table 2:** Sociodemographic characteristics of the respondents.

	Counts				Percent	
Gender						
Female	9				8%	
Male	102				92%	
Total	111				100%	
Experience practicing tumor-orthopedic surgery						
15 years or less	49				44%	
More than 15 years	62				56%	
Total	111				100%	
Region						
Europe	34				31%	
Americas	55				49%	
Asian Pacific	22				20%	
Total	111				100%	
	**Median**	**Mean**	**SD**	**Range**	**Missing values**	***n***
Age	51	50.2	10.4	31–77	3	111
Number of lower-extremity reconstruction limb surgery cases per year						
Non-oncological cases	45	82.2	92.3	0–350	5	111
Sarcoma cases	20	10.5	8.9	4–30	0	111

**Table 3:** Sports recommended; allowed, if asked; and not allowed after surgery.

pF/Hemi	pF/THR	dF	pT/ext. Ap	pT Composite
Sports recommended by at least 50% of respondents				
Walking	Walking	Walking	Walking	Walking
Swimming	Swimming	Swimming	Swimming	Swimming
Stationary bicycling	Stationary bicycling	Stationary bicycling	Stationary bicycling	Stationary bicycling
Golf	Low-impact aerobic	Golf	Golf	Hiking
		Low-impact aerobic		
Sports recommended or allowed, if asked by at least 50% of respondents				
Fishing	Fishing	Bowling	Fishing	Golf
Low-impact aerobics	Golf	Road bicycling	Low-impact aerobics	Fishing
Ballroom dancing	Low-impact aerobics	Hiking	Ballroom dancing	Ballroom dancing
Road bicycling	Ballroom dancing	Shuffleboard	Bowling	Bowling
Bowling	Bowling	Canoeing	Shuffleboard	Low-impact aerobics
Hiking	Hiking	Hunting	Hiking	Hiking
Yoga	Shuffleboard	Horseback riding	Yoga	Shuffleboard
Shuffleboard	Road bicycling	Rowing	Road bicycling	Road bicycling
Hunting	Yoga	Double tennis	Horseback riding	Yoga
Horseback riding	Horseback riding	Cross-country skiing	Canoeing	Horseback riding
Weight machines	Double tennis	Weight machines	Hunting	Canoeing
Canoeing	Hunting	Square dancing		Hunting
Rowing	Cross-country skiing			Cross-country skiing
Double tennis				Double tennis
Square dancing				
Cross-country skiing				
Sports not allowed by at least 50% of respondents				
Jazz dancing	Canoeing	Jazz dancing	Cross-country skiing	Rowing
Single tennis	Square dancing	Jogging	Rowing	Square dancing
Jogging	Rowing	Single tennis	Double tennis	Gymnastics
Gymnastics	Weight machines	Gymnastics	Weight machines	Weight machines
Weightlifting	Single tennis	Ice skating	Square dancing	Single tennis
Inline skating	Gymnastics	Inline skating	Gymnastics	Jazz dancing
Ice skating	Jazz dancing	Downhill skiing	Single tennis	Ice skating
Squash	Jogging	Squash	Jazz dancing	Jogging
Racquetball	Ice skating	Rock climbing	Jogging	Inline skating
Handball	Inline skating	High-impact aerobics	Ice skating	Racquetball
Downhill skiing	Squash	Racquetball	Inline skating	Downhill skiing
Rock climbing	Volleyball	Volleyball	Downhill skiing	Snowboarding
Volleyball	Downhill skiing	Weightlifting	Weightlifting	Handball
Baseball	Weightlifting	Baseball	High-impact aerobics	Squash
Snowboarding	High-impact aerobics	Basketball	Racquetball	Rock climbing
Basketball	Handball	Handball	Volleyball	Weightlifting
High-impact aerobics	Racquetball	Snowboarding	Handball	Volleyball
Soccer	Rock climbing	Soccer	Rock climbing	High-impact aerobics
Hockey	Basketball	Hockey	Basketball	Hockey
American football/Rugby	Baseball	American football/Rugby	Squash	Baseball
	Snowboarding		Baseball	Basketball
	Soccer		Snowboarding	Soccer
	Hockey		Soccer	American football/Rugby
	American football/Rugby		American football/Rugby	
			Hockey	

Table 3, sports recommended; allowed, if asked; and not allowed after surgery according to surgeons´ expert opinion. The sequence of the 40 sports corresponds to the average naming by participants according to recommended, recommended if asked, and not allowed. pF/Hemi = proximal femur megaprosthesis without acetabular replacement, pF/THR = proximal femur megaprosthesis with acetabular replacement as total hip replacement, dF = distal femur megaprosthesis, pT/ext. Ap = proximal tibia megaprosthesis with extensor apparatus reconstruction by gastrocnemius flap, pT = proximal tibia megaprosthesis as composite allograft prosthesis.

**Table 4:** Proportions (and 95% confidence intervals) of recommended, allowed, and not allowed sports after surgery by regions. Listed sports differed significantly between regions.

	Recommended	Allowed, if Asked	Not Allowed
Proximal femoral replacement hemiarthroplasty, monopolar or bipolar			
Golf			
Europe	42% (25%–61%)	58% (39%–75%)	0% (0%–14%)
Americas	71% (55%–84%)	29% (16%–45%)	0% (0%–10%)
Asian Pacific	29% (11%–56%)	53% (29%–76%)	18% (5%–44%)
Hunting			
Europe	6% (1%–23%)	68% (49%–83%)	26% (13%–45%)
Americas	14% (6%–29%)	71% (55%–84%)	14% (6%–29%)
Asian Pacific	6% (1%–31%)	24% (8%–50%)	71% (44%–89%)
Proximal femoral replacement THR			
Gymnastics			
Europe	19% (6%–43%)	52% (30%–74%)	29% (12%–52%)
Americas	0% (0%–15%)	31% (16%–51%)	69% (49%–84%)
Asian Pacific	0% (0%–24%)	13% (2%–40%)	88% (60%–98%)
Distal femoral replacement hinged TKR			
Stationary bicycling			
Europe	89% (70%–97%)	11% (3%–30%)	0% (0%–16%)
Americas	88% (72%–95%	13% (5%–28%)	0% (0%–11%)
Asian Pacific	43% (19%–70%)	43% (19%–17%)	14% (3%–44%)
Road bicycling			
Europe	59% (39%–77%)	30% (15%–50%)	11% (3%–32%)
Americas	25% (13%–42%)	65% (48%–79%)	10% (3%–25%)
Asian Pacific	7% (1%–34%)	60% (33%–83%)	33% (13%–61%
Proximal tibial replacement hinge TKR with soft tissue extensor reconstruction probable gastrocnemius rotation			
Gymnastics			
Europe	20% (8%–41%)	52% (32%–72%)	28% (13%–50%)
Americas	3% (1%–19%)	16% (6%–34%)	81% (62%–92%)
Asian Pacific	0% (0%–30%)	8% (1%–40%)	92% (60%–99%)

## Abbreviations

AAPOR RR1	American Association for Public Opinion Research First Response Rate
AAPOR RR2	American Association for Public Opinion Research Second Response Rate
dF	Distal femur megaprosthetic replacement
EMSOS	European Musculoskeletal Oncology Society
EU	European Union
ISOLS	International Society of Limb Salvage
MoReSports online survey	Modular Replacement Sports Survey
MSTS	Musculoskeletal Tumor Society
pF/ext.Ap	Proximal tibia megaprosthetic replacement with extensor apparatus reconstruction (e.g., gastrocnemius flap)
pF/Hemi	Proximal femur megaprosthesis without acetabular replacement
pF/THR	Proximal femur megaprosthesis with acetabular replacement
pT/composit	Proximal tibia megaprosthetic replacement with composite allograft prosthesis
QOL	Quality of life
TKR	Total knee replacement
UCLA Score	University of California Los Angeles Sports Activity Score
